# Commentary: Positive association between different triglyceride glucose index-related indicators and psoriasis: evidence from NHANES

**DOI:** 10.3389/fimmu.2025.1580061

**Published:** 2025-05-09

**Authors:** Bobiao Ning, Baohua Li, Ping Song

**Affiliations:** ^1^ Department of Dermatology, China Academy of Chinese Medical Sciences Guang’anmen Hospital, Beijing, China; ^2^ Eye Hospital, China Academy of Chinese Medical Sciences, Beijing, China; ^3^ Xiyuan Hospital, China Academy of Traditional Chinese Medicine, Beijing, China

**Keywords:** National Health and Nutrition Examination Survey (NHANES), psoriasis, triglyceride glucose-body mass index (TyG-BMI), triglyceride glucose-waist to height ratio (TyG-WHtR), and triglyceride glucose-waist circumference (TyG-WC)

## Introduction

We read with interest the article entitled “Positive correlation between different triglyceride glucose index-related indices and psoriasis: evidence from NHANES” by Huang et al. (1). This study utilized data from the National Health and Nutrition Examination Survey (NHANES) spanning 2003 to 2006 and 2009 to 2014, focusing on adults aged 20 to 80 years. It innovatively explored the relationship between psoriasis and triglyceride glucose–body mass index (TyG-BMI), triglyceride glucose–waist to height ratio (TyG-WHtR), and triglyceride glucose–waist circumference (TyG-WC). The study found that compared with the first quintile (Q1), TyG-BMI, TyG-WC, and TyG-WHtR of the second–third quintiles (Q2) and fourth–fifth quintiles (Q3) were significantly associated with psoriasis. Furthermore, the authors conducted subgroup analyses of the relationship between the three TyG indices and psoriasis based on all covariates, revealing that most covariates did not exhibit interaction effects. We acknowledge the valuable contributions made by the authors; however, we still have some questions regarding this study.

## Statistical methods

In the statistical analysis, the authors stated that they used multivariate logistic regression to examine the associations between the three TyG-related indices and psoriasis. However, since psoriasis is a binary outcome variable, it appears the authors may have conflated multivariate logistic regression with binary logistic regression. To enhance the rigor and accuracy of the study, we recommend revising the statistical analysis to specify the use of binary logistic regression. Additionally, the article notes that data with missing covariates were directly excluded. The authors should clarify the reasons for excluding participants due to missing covariates to ensure that selection bias was not introduced. In the subgroup analysis, the authors stated that there was no interaction between TyG-BMI and hypertension or hypertriglyceridemia (*p* for interaction > 0.05). However, this assertion appears to be inaccurate, as Figure 3 clearly demonstrates that the relationship between TyG-BMI and psoriasis is influenced by hypertension (*p* for interaction < 0.001). We believe this error should be corrected to prevent potential misinterpretation by readers.

## Covariate selection

The article has commendably incorporated covariates such as age, sex, race, education, smoking history, alcohol consumption, hypertension history, and diabetes history. However, given that numerous studies have shown physical activity can improve disease outcomes in patients with psoriasis (2), and that psoriasis imposes a long-term and substantial economic burden on patients and their families (3), we strongly recommend that the authors include physical activity and household income as additional covariates in their analysis.

## Assessment of predictive capacity

The final conclusion of this study states that TyG-related indices (TyG-BMI, TyG-WC, and TyG-WHtR) exhibit a stable and strong positive correlation with psoriasis. However, this conclusion may leave readers with the impression that the research is incomplete. For example, it remains unclear how these three TyG-related indices compare in terms of diagnostic capability for psoriasis. Therefore, we encourage the authors to incorporate a combined diagnostic test analysis to evaluate and compare the diagnostic performance of TyG-BMI, TyG-WC, and TyG-WHtR, which would be more helpful in guiding clinical practice.

## Discussion

Overall, this is a highly instructive study for clinical practice. To ensure that the research objectives are conveyed more accurately and comprehensively, we hope the authors will address the issues we have raised. We also believe this study will offer valuable insights into the prevention and management of psoriasis.
